# Long-Term Outcomes After Transcatheter Aortic Valve Replacement in Nonagenarians: Impact of Frailty and Malnutrition

**DOI:** 10.1016/j.jacadv.2026.102614

**Published:** 2026-03-25

**Authors:** Tetsuya Kobayashi, Masahiko Noguchi, Masanori Yamamoto, Joji Ito, Nahoko Kato, Makio Muraishi, Kunihiko Yoshino, Hiroki Sakai, Tetsuro Shimura, Hirofumi Hioki, Shinichi Shirai, Kenichi Ishizu, Yohei Ohno, Fumiaki Yashima, Toru Naganuma, Yusuke Watanabe, Futoshi Yamanaka, Gaku Nakazawa, Masaki Izumo, Masahiko Asami, Hidetaka Nishina, Yasushi Fuku, Toshiaki Otsuka, Kentaro Hayashida, OCEAN-TAVI Investigators

**Affiliations:** a Department of Cardiology, Tokyo Bay Urayasu Ichikawa Medical Center, Urayasu, Japan; b Department of Cardiology, Toyohashi Heart Center, Toyohashi, Japan; c Department of Cardiology, Nagoya Heart Center, Nagoya, Japan; d Department of Cardiology, Gifu Heart Center, Gifu, Japan; e Department of Cardiovascular Surgery, Tokyo Bay Urayasu Ichikawa Medical Center, Uyarasu, Japan; f Department of Cardiology, IMS Tokyo Katsushika General Hospital, Tokyo, Japan; g Department of Cardiology, Kokura Memorial Hospital, Kitakyushu, Japan; h Department of Cardiology, Tokai University School of Medicine, Isehara, Japan; i Department of Cardiology, Saiseikai Utsunomiya Hospital, Utsunomiya, Japan; j Department of Cardiology, New Tokyo Hospital, Matsudo, Japan; k Department of Cardiology, Teikyo University School of Medicine, Tokyo, Japan; l Department of Cardiology, Shonan Kamakura General Hospital, Kamakura, Japan; m Department of Cardiology, Kindai University, Osaka, Japan; n Department of Cardiology, St. Marianna University School of Medicine, Kawasaki, Japan; o Division of Cardiology, Mitsui Memorial Hospital, Tokyo, Japan; p Department of Cardiology, Tsukuba Medical Center Hospital, Tsukuba, Japan; q Department of Cardiovascular Medicine, Kurashiki Central Hospital, Okayama, Japan; r Department of Hygiene and Public Health, Nippon Medical School, Tokyo, Japan; s Center for Clinical Research, Nippon Medical School Hospital, Tokyo, Japan; t Department of Cardiology, Keio University School of Medicine, Tokyo, Japan

**Keywords:** frailty, long-term outcomes, malnutrition, nonagenarians, transcatheter aortic valve replacement

## Abstract

**Background:**

The role of transcatheter aortic valve replacement (TAVR) in nonagenarians remains uncertain, especially regarding long-term outcomes and prognostic factors.

**Objectives:**

This study aimed to evaluate long-term outcomes of TAVR in nonagenarians, focusing on cause-specific mortality and the prognostic influence of frailty and malnutrition.

**Methods:**

We analyzed 4,623 patients who underwent transfemoral TAVR in a multicenter Japanese registry, including 700 aged ≥90 years. Outcomes were followed for 5 years. We analyzed all-cause mortality, cause-specific mortality, and the prognostic impact of the Clinical Frailty Scale and Geriatric Nutritional Risk Index.

**Results:**

At 5 years, all-cause mortality was higher in patients aged ≥90 years than in those <90 years (53.2% vs37.0%, *P* < 0.001), primarily due to noncardiovascular deaths such as senility and infections (32.5% vs 19.9%; *P* < 0.001). Cardiovascular mortality was similar (20.3% vs 17.0%; *P* = 0.198). Multivariable analysis showed that age ≥90 years was not an independent predictor; frailty and malnutrition were the strongest prognostic factors. A Clinical Frailty Scale–Geriatric Nutritional Risk Index heatmap revealed marked heterogeneity, identifying subgroups of nonagenarians with preserved nutrition and low frailty who achieved favorable long-term survival.

**Conclusions:**

In this large multicenter registry, excess mortality in nonagenarians after TAVR was driven mainly by noncardiovascular causes. Frailty and malnutrition, rather than chronological age, were central determinants of long-term outcomes. These findings emphasize that TAVR candidacy in nonagenarians should be guided on geriatric assessment of frailty and nutrition to identify patients most likely to achieve meaningful survival while avoiding futile interventions.

Severe aortic stenosis is increasingly diagnosed in older adults,^1^ and transcatheter aortic valve replacement (TAVR) is now an established therapy across a broad spectrum of surgical risk.^2^^,^^3^ In the oldest patients, particularly nonagenarians (aged ≥90 years), the decision to pursue TAVR remains complex because of limited life expectancy and competing risks. Prior studies have shown acceptable short-term safety in this population;^4^^,^^5^ however, long-term outcomes and specific causes of death remain insufficiently defined. Clarifying the factors driving late mortality in this cohort is therefore crucial.

Among potential determinants, geriatric characteristics warrant special consideration, as patients undergoing TAVR at advanced age often have reduced physiologic reserve.^6^ Frailty and malnutrition, although related, are distinct and well-established prognostic markers.^7^^,^^8^ However, their specific impact in nonagenarians has not been systematically evaluated.

Accordingly, we used a large multicenter Japanese registry with extended follow-up to: 1) characterize long-term outcomes and cause-specific mortality after TAVR in patients aged ≥90 years; and 2) determine the prognostic significance of frailty and nutritional status. We hypothesized that excess mortality in nonagenarians would be primarily attributable to noncardiovascular causes and that frailty and malnutrition would be key predictors of long-term prognosis.

## Methods

### Study population

This study used data from the Optimized transCathEter vAlvular intervention aortic valve implantation registry, a prospective multicenter registry of patients undergoing TAVR in Japan. The registry is listed with the University Hospital Medical Information Network Clinical Trials Registry (UMIN000020423). Protocol approval was obtained from institutional ethics committees at all centers in accordance with the Declaration of Helsinki. A written informed consent was obtained from all patients. Between October 2013 and September 2019, 5,237 patients underwent TAVR at participating institutions. A total of 614 patients were excluded: 9 aged <60 years, 4 receiving chronic hemodialysis, 551 who underwent nontransfemoral approaches, and 50 who underwent emergency procedures. To maintain a homogeneous cohort, only transfemoral cases were analyzed. The final population included 4,623 patients aged ≥60 years, stratified into nonagenarians (≥90 years, n = 700) and patients aged <90 years (n = 3,923). A study flow diagram is shown in Supplemental Figure 1.

### Study endpoints

The primary endpoint was all-cause mortality after TAVR. Secondary endpoints included cause-specific mortality (cardiovascular and noncardiovascular), hospitalization for heart failure, and stroke. Causes of death were adjudicated by the treating physicians at each site and classified using Valve Academic Research Consortium-3 definitions.^9^ For this analysis, deaths attributed to senility (old age) were categorized as noncardiovascular and pneumonia was classified as infection, consistent with Japanese death certificate conventions.

### Frailty and nutritional status

Frailty was assessed with the Clinical Frailty Scale (CFS), based on the Canadian Study of Health and Aging criteria.^10^^,^^11^ The CFS ranges from 1 (very fit) to 9 (terminally ill) and was determined at each center before TAVR.

Nutritional status was evaluated using the Geriatric Nutritional Risk Index (GNRI), a validated marker of malnutrition in older adults.^12^ GNRI was calculated as:

$$\text{GNRI}=1.489\times \text{serum}\;\text{albumin}\;\left(g/L\right)+41.7\times \frac{\text{current}\;\text{body}\;\text{weight}\;\left(\text{kg}\right)}{\text{ideal}\;\text{body}\;\text{weight}\;\left(\text{kg}\right)}$$

where the ideal body weight was defined as 22 × height^2^ (m^2^). If the current body weight exceeded the ideal body weight, the ratio was set to 1.

### Statistical analysis

Continuous variables are expressed as median (IQR) and compared using the Wilcoxon rank-sum test. Categorical variables are presented as counts (percentages) and compared using the chi-square or Fisher exact test. Survival was calculated from the index TAVR procedure. All-cause mortality was estimated using the Kaplan-Meier method and compared with the log-rank test. Cause-specific outcomes were analyzed with cumulative incidence functions in a competing-risks framework (Fine-Gray method). Cardiovascular and noncardiovascular deaths were treated as reciprocal competing risks, and all deaths were considered competing events for nonfatal outcomes. Group differences were tested with the Gray test. Event rates were estimated at 30 days, 1 year, and 5 years. The median follow-up duration was estimated using the reverse Kaplan-Meier method.

Multivariable models included Cox proportional hazards for all-cause mortality and Fine-Gray subdistribution hazard models for cause-specific outcomes. Prespecified covariates were age, sex, NYHA functional class III–IV, left ventricular ejection fraction, CFS, GNRI, chronic kidney disease, atrial fibrillation (AF), diabetes mellitus, and chronic obstructive pulmonary disease. Analyses were limited to complete cases; 58 patients (1.25%) with missing covariates were excluded. Results are reported as HRs or subdistribution HRs (sHRs) with 95% CIs.

In addition, a prespecified sensitivity analysis compared patients aged ≥90 years with those aged 80 to 89 years using the same multivariable adjustment to evaluate the robustness of the primary findings.

Collinearity between CFS and GNRI was evaluated with Pearson and Spearman correlation coefficients and variance inflation factors (VIFs); |r| ≤0.5 and VIF <5 indicated no significant collinearity. Exploratory analyses included assessment of prognostic factors in nonagenarians, spline modeling of age as a continuous variable, and comparison of observed survival with age- and sex-matched expected survival from Japanese life tables^13^ using the Ederer II method. All analyses were 2-sided, with *P* < 0.05 considered statistically significant. Analyses were performed with R (version 4.2.2, R Foundation for Statistical Computing).

## Results

### Baseline characteristics

Of 5,237 patients who underwent TAVR during the study period, 614 were excluded, leaving 4,623 in the final cohort. This included 700 (15.1%) aged ≥90 years and 3,923 aged <90 years. Baseline characteristics are summarized in Table 1. Compared with younger patients, nonagenarians were more frequently females; had smaller body size; and demonstrated higher rates of frailty (CFS ≥4: 68.0% vs 53.9%), malnutrition (median GNRI: 93.0 vs 96.8), and chronic kidney disease (81.6% vs 68.8%). In contrast, diabetes mellitus and dyslipidemia were less prevalent. Left ventricular ejection fraction did not differ between groups.

**Table 1:** Baseline Characteristics of the Study Populations

	Age ≥90 years (n = 700)	Age <90 years (n = 3,923)	*P* Value
Age, y	91 (90-93)	84 (81-86)	<0.001
Female	529 (75.6%)	2,671 (68.1%)	<0.001
BSA, m^2^	1.34 (1.25-1.45)	1.44 (1.32-1.57)	<0.001
BMI, kg/m^2^	21.0 (18.7-23.3)	22.2 (19.8-24.6)	<0.001
Clinical Frailty Scale	4 (3-4)	4 (3-5)	<0.001
Clinical Frailty Scale ≥4	474 (68.0%)	2094 (53.9%)	<0.001
Clinical Frailty Scale ≥5	235 (33.7%)	834 (21.5%)	<0.001
Clinical Frailty Scale ≥6	101 (14.5%)	380 (9.8%)	<0.001
GNRI	93.0 (87.0-98.3)	96.8 (90.6-101.3)	<0.001
GNRI≤98	505 (72.5%)	2,213 (56.7%)	<0.001
GNRI≤92	310 (44.5%)	1,165 (29.8%)	<0.001
GNRI≤82	94 (13.5%)	316 (8.1%)	<0.001
NYHA functional class III/IV	343 (49.2%)	1,482 (38.0%)	<0.001
Current smoker	5 (0.7%)	56 (1.4%)	0.031
Hypertension	609 (87.0%)	3,311 (84.4%)	0.088
Diabetes mellitus	140 (20.0%)	1,193 (30.4%)	<0.001
Dyslipidemia	353 (50.4%)	2,282 (58.2%)	<0.001
Atrial fibrillation	178 (25.5%)	876 (22.3%)	0.077
eGFR, mL/min/1.73 m^2^	45.2 (34.5-57.0)	51.3 (39.3-63.6)	<0.001
CKD	571 (81.6%)	2,700 (68.8%)	<0.001
Prior PCI	127 (18.1%)	938 (23.9%)	0.001
Prior CABG	20 (4.6%)	175 (6.9%)	0.085
Prior MI	21 (3.0%)	217 (5.5%)	0.007
Prior stroke	74 (10.6%)	473 (12.1%)	0.294
Peripheral artery disease	98 (14.0%)	366 (9.3%)	<0.001
COPD	64 (9.2%)	380 (9.7%)	0.712
Previous PM/ICD	62 (8.9%)	209 (5.3%)	<0.001
STS PROM score	9.34 (6.85-12.6)	5.51 (4.00-8.08)	<0.001
Echocardiographic characteristics			
AVA, cm^2^	0.58 ± 0.17	0.64 ± 0.19	<0.001
Indexed AVA, cm^2^/m^2^	0.43 ± 0.12	0.44 ± 0.13	0.001
AV Vmax, m/s	4.6 ± 0.8	4.5 ± 0.8	0.008
AV PG mean, mm Hg	50.7 ± 18.4	48.6 ± 18.1	0.006
SVI, mL/m^2^	44.5 ± 13.0	45.7 ± 12.9	0.036
Low flow/low gradient	67 (9.6%)	370 (9.4%)	0.963
LVEF, %	60.0 ± 12.0	60.9 ± 12.0	0.078
LVEF <40%	58 (8.3%)	295 (7.0%)	0.533
SPAP, mm Hg	34.4 ± 11.7	32.5 ± 11.4	<0.001
Moderate ≤ AR	67 (9.6%)	436 (11.1%)	0.25
Moderate ≤ MR	116 (16.6%)	496 (12.6%)	0.006
Procedural characteristics			
Valve type			0.364
Sapien XT	111 (15.9%)	691 (17.6%)	
Sapien 3	390 (55.7%)	2,246 (57.3%)	
CoreValve	25 (3.6%)	135 (3.4%)	
Evolut R	115 (16.4%)	580 (14.8%)	
Evolut Pro	59 (8.4%)	271 (6.9%)	
Valve Size, mm			0.001
20	44 (6.3%)	153 (3.9%)	
23	346 (49.4%)	1814 (46.2%)	
26	247 (35.3%)	1,441 (36.7%)	
29	63 (9.0%)	515 (13.1%)	

Values are n (%) or median (IQR).

AR = aortic regurgitation; AVA = aortic valve area; AV PG mean = aortic valve mean pressure gradient; AV Vmax = aortic valve maximum velocity; BMI = body mass index; BSA = body surface area; CABG = coronary artery bypass grafting; CKD = chronic kidney disease; COPD = chronic obstructive pulmonary disease; eGFR = estimated glomerular filtration rate; GNRI = Geriatric Nutritional Risk Index; ICD = implantable cardioverter-defibrillator; LVEF = left ventricular ejection fraction; MI = myocardial infarction; MR = mitral regurgitation; PCI = percutaneous coronary intervention; PM = pacemaker; SPAP = systolic pulmonary artery pressure; STS PROM = Society of Thoracic Surgeons predicted risk of operative mortality; SVI = stroke volume index.

### Long-term outcomes

**Figure 1:**
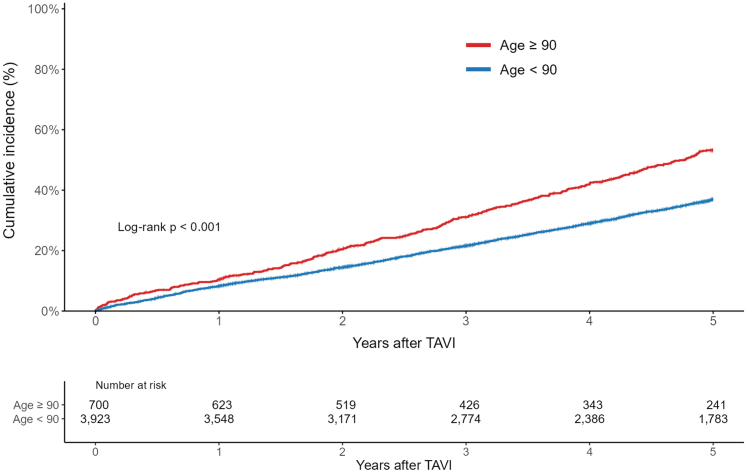
**Cumulative Incidence of All-Cause Death After Transcatheter Aortic Valve Replacement by Age Group** Kaplan-Meier curves compare patients aged ≥90 years (red) with those aged <90 years (blue). Numbers at risk are displayed annually. The curve for nonagenarians consistently lies above that for younger patients, with progressive separation (log-rank *P* < 0.001). TAVI = transcatheter aortic valve implantation; TAVR = transcatheter aortic valve replacement.

**Figure 2:**
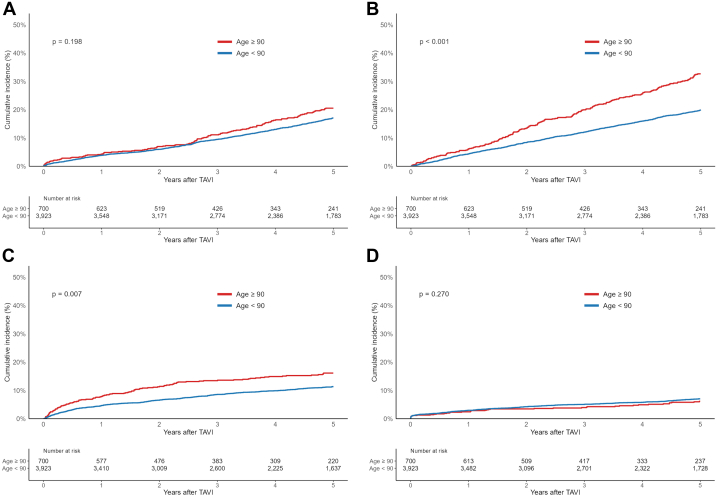
**Cumulative Incidence of Outcomes by Age Group** Cumulative incidence functions are shown for (A) cardiovascular death, (B) noncardiovascular death, (C) heart failure readmission, and (D) stroke, stratified by age (red: ≥90 years; blue: <90 years). *P* values were calculated using Gray test. At 5 years, noncardiovascular death and heart failure readmission were significantly higher in patients aged ≥90 years, whereas cardiovascular death and stroke were not significantly different between groups. Abbreviation as in Figure 1.

**Figure 3:**
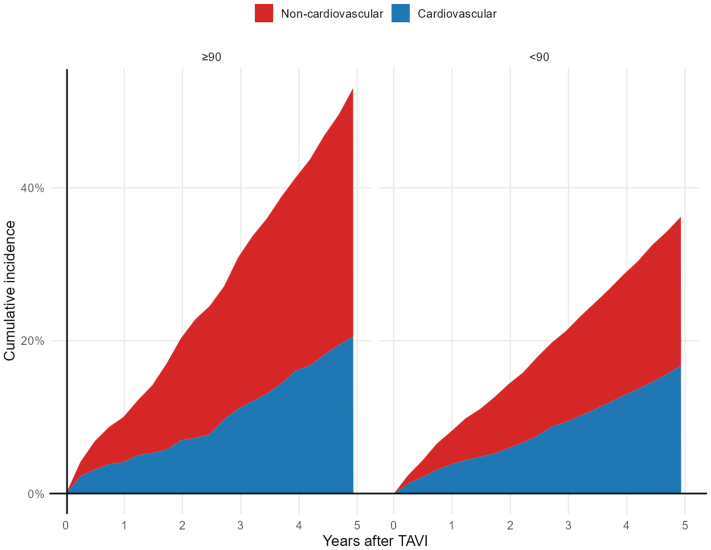
**Stacked Cumulative Incidence of Noncardiovascular and Cardiovascular Deaths** Stacked cumulative incidence functions are shown for noncardiovascular and cardiovascular deaths, stratified by age group (left: ≥90 years, right: <90 years). The between-group difference is largely explained by noncardiovascular deaths, whereas cardiovascular deaths accumulate at similar rates across groups. Noncardiovascular and cardiovascular deaths are displayed in red and blue, respectively. Abbreviation as in Figure 1.

Median follow-up was 5.6 years (IQR: 5.0-7.0 years). At 5 years, all-cause mortality was significantly higher in nonagenarians compared with younger patients (53.2%; 95% CI: 49.1%-57.0% vs 37.0%; 95% CI: 35.4%-38.6%; log-rank *P* < 0.001) (Figure 1). Cause-specific incidence indicated that the excess risk was driven mainly by noncardiovascular death (32.5%; 95% CI: 28.8%-36.1% vs 19.9%; 95% CI: 18.6%-21.3%; *P* < 0.001), whereas cardiovascular mortality was similar (20.3%; 95% CI: 17.2%-23.4% vs 17.0%; 95% CI: 15.8%-18.3%; *P* = 0.198) (Figures 2A and 2B). The 5-year cumulative incidence of heart failure readmission was also higher in nonagenarians (15.9%; 95% CI: 13.2%-18.6% vs 11.3%; 95% CI: 10.3%-12.4%; *P* = 0.007), whereas stroke incidence did not differ (6.0%; 95% CI: 4.2%-7.7% vs 7.0%; 95% CI: 6.2%-7.9%; *P* = 0.270) (Figures 2C and 2D). Estimated event rates at 30 days and 1 year, and 5 years for all endpoints are provided in Table 2. Stacked cumulative incidence curves revealed that the between-group difference in all-cause mortality was explained almost entirely by noncardiovascular causes (Figure 3). At 5 years, the absolute risk difference (≥90 minus <90) was +16.0 percentage points for noncardiovascular death and +0.7 for cardiovascular death.

**Table 2:** Event Rates by Age Group After TAVR (≥90 vs <90)

Endpoint	30 Days (≥90 vs <90)	1 Year (≥90 vs <90)	5 Years (≥90 vs <90)	*P* Value
All-cause death	2.1% vs 1.1%	10.5% vs 8.2%	53.2% vs 37.0%	<0.001
Cardiovascular death	1.3% vs 0.7%	4.2% vs 3.8%	20.3% vs 17.0%	0.198
Noncardiovascular death	0.6% vs 0.3%	5.9% vs 4.4%	32.5% vs 19.9%	<0.001
Heart failure readmission	1.1% vs 0.6%	7.7% vs 4.6%	15.9% vs 11.3%	0.007
Stroke	1.0% vs 1.2%	2.3% vs 2.9%	6.0% vs 7.0%	0.270

All-cause mortality was estimated using Kaplan-Meier methods, whereas other endpoints were estimated using cumulative incidence functions. *P* values compare age ≥90 vs <90 (log-rank for all-cause mortality; Gray test for other endpoints).

TAVR = transcatheter aortic valve replacement.

### Adjusted outcomes

In multivariable models, age ≥90 years was not independently associated with all-cause mortality (HR: 1.10; 95% CI: 0.95-1.27; *P* = 0.186), cardiovascular death (sHR: 0.91; 95% CI: 0.73-1.14; *P* = 0.432), noncardiovascular death (sHR: 1.19; 95% CI: 0.98-1.45; *P* = 0.083), or heart failure readmission (sHR: 1.22; 95% CI: 0.93-1.60; *P* = 0.156). A lower stroke risk was observed in nonagenarians, although this was borderline significant (sHR: 0.68; 95% CI: 0.46-1.00; *P* = 0.047) (Table 3). A sensitivity analysis comparing ≥90 vs 80 to 89 years showed largely similar results to the primary analysis (Supplemental Table 1). CFS and GNRI were modestly correlated (r ≈ –0.3), with all VIF values <1.3, excluding collinearity.

**Table 3:** Adjusted Associations Between Age ≥90 Years and Post-TAVR Outcomes

Endpoint	Unadjusted HR (95% CI)	*P* Value	Adjusted HR (95% CI)	*P* Value
All-cause death	1.58 (1.42-1.76)	<0.001	1.10 (0.95-1.27)	0.186
Cardiovascular death	1.12 (0.94-1.33)	0.193	0.91 (0.73-1.14)	0.432
Noncardiovascular death	1.71 (1.49-1.97)	<0.001	1.19 (0.98-1.45)	0.083
Hear failure readmission	1.34 (1.09-1.65)	0.005	1.22 (0.93-1.60)	0.156
Stroke	0.84 (0.62-1.16)	0.290	0.68 (0.46-1.00)	0.047

Abbreviations as in Table 2.

### Prognostic factors in nonagenarians

**Figure 4:**
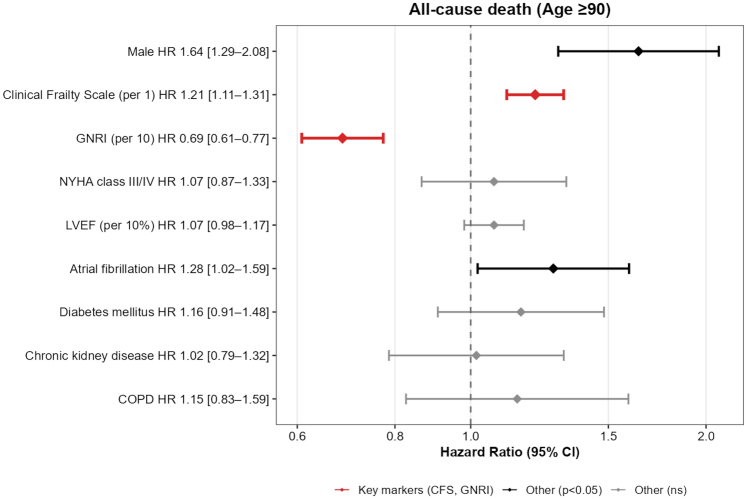
**Multivariable Predictors of All-Cause Death in Nonagenarians After Transcatheter Aortic Valve Replacement** Forest plot of HRs with 95% CIs from a Cox proportional hazards model including prespecified covariates: male sex, Clinical Frailty Scale (per 1-point increment), GNRI (per 10-point increment), NYHA functional class III/IV, LVEF (per 10% increase), atrial fibrillation, diabetes mellitus, chronic kidney disease, and COPD. Red markers indicate key geriatric factors (frailty and nutrition), black markers significant predictors (*P* < 0.05), and gray markers nonsignificant predictors. CFS = Clinical Frailty Scale; COPD = chronic obstructive pulmonary disease; GNRI = Geriatric Nutritional Risk Index; LVEF = left ventricular ejection fraction.

**Figure 5:**
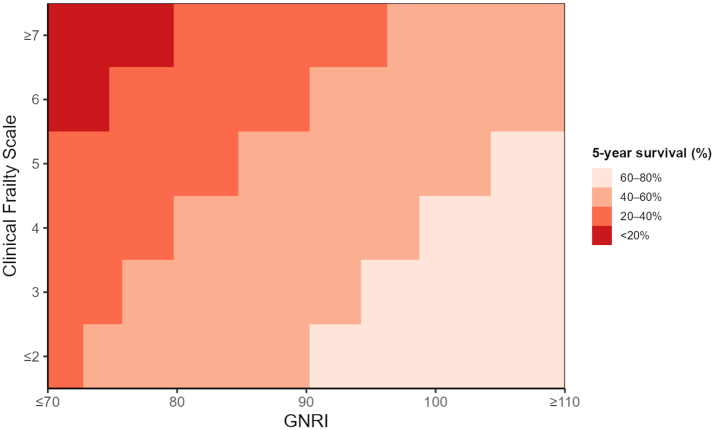
**5-Year Survival by Frailty and Nutrition Among Nonagenarians After Transcatheter Aortic Valve Replacement** A multivariable Fine-Gray model was used to estimate 5-year overall survival among nonagenarians after TAVR. The heatmap illustrates survival across GNRI (≤70 to ≥110) and CFS (≤2 to ≥7), with other covariates fixed at cohort medians. Lighter shades correspond to higher survival (60-80%), whereas darker shades indicate lower survival (20%-40% or <20%). A clear gradient is observed from low GNRI/high frailty (worst prognosis) to high GNRI/low frailty (best prognosis). Abbreviations as in Figure 4.

Among patients aged ≥90 years, frailty and nutritional status were the strongest prognostic markers. Each 1-point increase in CFS was associated with a 21% higher mortality risk (HR: 1.21; 95% CI: 1.11-1.31), whereas each 10-point increase in GNRI conferred a 31% lower mortality risk (HR: 0.69; 95% CI: 0.61-0.77) (Figure 4). Male sex (HR: 1.64; 95% CI: 1.29-2.08) and AF (HR 1.28; 95% CI: 1.02-1.59) were also independently associated with worse prognosis. A heatmap combining CFS and GNRI (Figure 5) showed marked heterogeneity: nonagenarians with high frailty (CFS ≥6) and low GNRI (≤70) had <20% 5-year survival, whereas those with low frailty (CFS ≤3) and high GNRI (≥100) exceeded 60% survival.

### Exploratory analyses

Exploratory analyses are detailed in the Supplement (Supplemental Figures S2 to S4). Briefly, age-spline modeling revealed a gradual increase in mortality without a discrete threshold at 90 years. Cause-of-death subcategorization confirmed higher proportions of senility and infection among nonagenarians. Relative survival suggested that, beyond the early hazard phase, survival in some nonagenarians approximated that of the age- and sex-matched general population.

## Discussion

In this large multicenter registry, we evaluated long-term outcomes of TAVR in patients aged ≥90 years. Several key findings emerged (Central Illustration). First, nonagenarians had significantly higher all-cause mortality than younger patients; however, this excess risk was driven almost entirely by noncardiovascular causes, whereas cardiovascular mortality was comparable across age groups. Second, after adjusting for comorbidities and geriatric domains, chronological age ≥90 years was not an independent predictor of adverse outcomes. Instead, frailty and malnutrition were the strongest prognostic factors for survival. Third, stratification by CFS and GNRI revealed considerable heterogeneity among nonagenarians, with a subset achieving survival comparable to younger cohorts. These results indicate that patients aged ≥90 years should not be denied TAVR solely on the basis of chronological age, but should instead be evaluated in the context of biological age. Prior studies have reported poorer outcomes with advanced age after TAVR; however, accumulating evidence suggests that this association is largely driven by baseline vulnerability rather than chronological age itself. Large registries, including the STS/ACC TVT Registry and the CENTER collaboration, demonstrated higher crude mortality among nonagenarians, yet adjusted analyses consistently showed that age ≥90 years was not an independent predictor of adverse outcomes, with frailty and comorbidity burden accounting for much of the excess risk.^4^^,^^5^ Consistent with these observations, our prior analysis from the Optimized transCathEter vAlvular intervention aortic valve implantation registry showed that age ≥90 years was not independently associated with 2-year mortality, whereas frailty and renal dysfunction were key prognostic factors.^14^ In addition, the OBSERVANT II study, a frailty-focused subanalysis conducted across a broad age range, demonstrated that the coexistence of functional and nutritional frailty was associated with substantially worse survival, reinforcing the prognostic importance of geriatric vulnerability beyond chronological age.^15^ More recently, Delijani et al^16^, using a large U.S. administrative database, demonstrated that older adults experienced higher rates of complications, mortality, and readmission, with frailty emerging as the major determinant of prognosis rather than comorbidity burden. Taken together, these international data place our findings in a broader context and support the concept that geriatric vulnerability—particularly frailty and nutritional status—rather than chronological age per se, is the principal driver of long-term outcomes after TAVR.

**Central Illustration:**
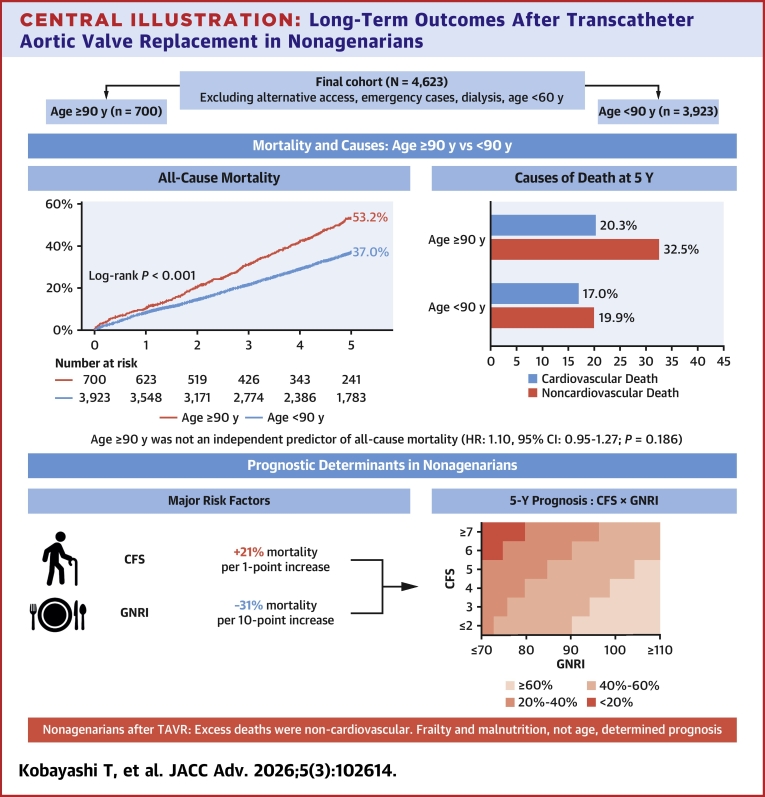
**Long-Term Outcomes After Transcatheter Aortic Valve Replacement in Nonagenarians** Nonagenarians undergoing TAVR had higher long-term mortality driven predominantly by noncardiovascular causes. Chronological age ≥90 years was not an independent predictor of outcomes; instead, frailty and malnutrition were the principal determinants of long-term prognosis. CFS = Clinical Frailty Scale; GNRI = Geriatric Nutritional Risk Index; TAVR = transcatheter aortic valve replacement.

Importantly, our study extends these prior observations by providing up to 5-year follow-up data. Excess mortality in nonagenarians was largely explained by noncardiovascular causes, such as senility and infections, whereas frailty and malnutrition remained dominant prognostic factors throughout long-term follow-up. Evidence on heart failure readmission in nonagenarians after TAVR has been limited.^14^ In our analysis, crude rates of heart failure readmission were higher in nonagenarians; however, this difference was no longer evident after adjustment, suggesting that TAVR may mitigate excess risk of heart failure progression in this age group. Stroke incidence remained low and comparable between age groups, consistent with earlier reports.^4^^,^^5^

The predominance of noncardiovascular deaths, particularly senility and infections, aligns with the biological vulnerability associated with frailty and malnutrition. These domains reflect reduced physiological reserve, leading to functional decline and immune aging, ultimately resulting in dementia-related and infectious deaths.^17^, ^18^, ^19^ This provides a mechanistic explanation for cause-specific mortality, emphasizing that geriatric vulnerabilities, rather than cardiac disease progression, drive long-term outcomes. Indeed, large TAVR registries have shown that frailty and malnutrition are each independently associated with adverse long-term outcomes.^7^^,^^20^ Frailty and malnutrition, although partially overlapping, are distinct: frailty reflects functional reserve, whereas malnutrition reflects metabolic reserve. Prior studies showed only modest correlation between the 2, with each adding prognostic value.^8^ The OBSERVANT II study further showed that their coexistence doubled mortality risk.^15^ Building on this evidence, our heatmap analysis demonstrated that nonagenarians with high frailty (CFS ≥6) and low GNRI (≤70) had <20% 5-year survival, whereas those with low frailty (CFS ≤3) and high GNRI (≥100) achieved >60% survival. Thus, the dual assessment of frailty and nutrition refines risk stratification beyond both factors alone and supports integration into clinical decision-making in advanced age. Furthermore, long-term survival in selected nonagenarians compared favorably with age- and sex-matched life expectancy in the general Japanese population, consistent with earlier reports.^21^

In addition, male sex and AF—factors previously associated with adverse outcomes after TAVR—were also associated with higher mortality among nonagenarians, suggesting that conventional clinical risk markers may complement geriatric domains in refining long-term risk stratification even in very advanced age.^4^^,^^22^

Overall, these findings highlight substantial heterogeneity in outcomes among appropriately selected nonagenarians undergoing TAVR, and that chronological age ≥90 years should not be considered an absolute contraindication. Instead, comprehensive geriatric assessment—including frailty and nutritional status—should guide patient selection to identify those most likely to achieve meaningful survival while avoiding futile interventions. In this context, our results support incorporating standardized frailty and nutritional assessments into routine pre-TAVR evaluation in very old adults. CFS and GNRI are simple, readily available measures of functional and metabolic reserve and may help identify patients unlikely to derive meaningful benefit from TAVR. A recent American Heart Association scientific statement emphasized frailty—including malnutrition and functional decline—as an independent and potentially modifiable determinant of cardiovascular outcomes and recommended routine frailty assessment in older adults.^23^ In line with this guidance, future studies should determine whether targeted strategies, such as prehabilitation and nutrition-focused interventions can enhance physiological resilience and improve long-term outcomes in nonagenarians undergoing TAVR.

### Study limitations

This study has several limitations. First, classification of cause of death relied on site-reported data and may be subject to misclassification. In particular, “senility” on death certificates, although common in Japanese clinical practice, lacks universal standardization and may limit international comparability. Second, as with all observational studies, residual confounding cannot be excluded despite multivariable adjustment. Because frailty and nutritional status are closely related to aging and may lie on the causal pathway between chronological age and adverse outcomes, adjustment for these variables may have attenuated the apparent effect of age itself. Third, the present cohort represents a selected population of nonagenarians deemed suitable for transfemoral TAVR after multidisciplinary evaluation. As only patients who underwent TAVR were included, these nonagenarians likely constitute a relatively fitter subset with favorable vascular anatomy and sufficient physiological reserve, and our findings may not be fully generalizable to all patients aged ≥90 years with severe aortic stenosis, particularly those managed conservatively, deemed unsuitable for intervention, or requiring alternative access routes. Fourth, frailty and nutritional status were assessed using CFS and GNRI, both of which have inherent limitations. CFS is subjective and may be subject to intercenter variability, and GNRI—calculated using ideal body weight defined as 22 × height^2^, the standard method in Japan—assumes a uniform ideal body mass index and does not account for sex-specific differences, raising the possibility of misclassification. Finally, because this study was conducted in a Japanese cohort and included patients treated with earlier-generation TAVR devices, caution is warranted when extrapolating our findings to other populations or to contemporary TAVR practice. Nevertheless, in the context of global population aging, evidence from Japan—where longevity and the prevalence of very old patients are among the highest—offers valuable insights likely to be broadly relevant.

## Conclusions

In summary, patients aged ≥90 years undergoing TAVR had higher long-term all-cause mortality than younger patients, primarily due to noncardiovascular causes. Chronological age was not an independent predictor of outcomes; instead, frailty and malnutrition were the principal determinants of survival. Accordingly, patient selection for TAVR in nonagenarians should be guided by geriatric assessment focused on frailty and nutrition status to identify individuals likely to achieve meaningful survival while avoiding futile interventions.

Perspectives **COMPETENCY IN MEDICAL KNOWLEDGE:** Among patients aged ≥90 years undergoing TAVR, excess long-term mortality is driven predominantly by noncardiovascular causes rather than cardiovascular death. Chronological age alone is not an independent predictor of outcomes; instead, frailty and nutritional status are the principal determinants of long-term prognosis. Simple geriatric assessments, including the CFS and the GNRI, provide clinically relevant prognostic information beyond age in nonagenarians.

**TRANSLATIONAL OUTLOOK:** Future studies should evaluate whether systematic incorporation of frailty and nutritional assessment into pre-TAVR evaluation improves patient selection and outcomes in nonagenarians. In addition, interventional strategies targeting potentially modifiable geriatric vulnerabilities, including prehabilitation and nutritional optimization, warrant investigation to enhance physiological resilience and long-term outcomes in this growing population.

## Funding support and author disclosures

The OCEAN-TAVI registry is supported by 10.13039/100006520Edwards-Lifesciences, 10.13039/100004374Medtronic, 10.13039/100011949Abbott Vascular, 10.13039/100008497Boston Scientific, and 10.13039/501100002973Daiichi-Sankyo. The sponsors had no role in study design, data collection, analysis, or manuscript preparation. Dr Ito is a clinical proctor for 10.13039/100006520Edwards Lifesciences. Dr Izumo is a screening proctor for 10.13039/100006520Edwards Lifesciences. Dr Ishizu is a proctor of intracardiac echocardiography for 10.13039/100004331Johnson and Johnson. Drs Yashima and Nishina are clinical proctors for 10.13039/100004374Medtronic. Drs Ohno and Shimura are clinical proctors for 10.13039/100004374Medtronic and Abbott Medical. Drs Yamamoto, Shirai, Naganuma, Watanabe, Nakazawa, Asami, Fuku, and Hayashida served as clinical proctors for 10.13039/100006520Edwards Lifesciences, Abbott Medical, and 10.13039/100004374Medtronic. All other authors have reported that they have no relationships relevant to the contents of this paper to disclose.

## References

[1] Danielsen R., Aspelund T., Harris T.B., Gudnason V. (2014). The prevalence of aortic stenosis in the elderly in Iceland and predictions for the coming decades: the AGES-Reykjavík study. Int J Cardiol.

[2] Otto C.M., Nishimura R.A., Bonow R.O. (2021). 2020 ACC/AHA guideline for the management of patients with valvular heart disease: a report of the American College of Cardiology/American Heart Association joint committee on clinical practice guidelines. J Am Coll Cardiol.

[3] Praz F., Borger M.A., Lanz J. (2025). 2025 ESC/EACTS guidelines for the management of valvular heart disease. Eur Heart J.

[4] Arsalan M., Szerlip M., Vemulapalli S. (2016). Should transcatheter aortic valve replacement be performed in nonagenarians?: insights from the STS/ACC TVT registry. J Am Coll Cardiol.

[5] Vlastra W., Chandrasekhar J., Vendrik J. (2019). Transfemoral TAVR in nonagenarians: from the CENTER collaboration. JACC Cardiovasc Interv.

[6] Damluji A.A., Bernacki G., Afilalo J. (2024). TAVR in older adults: moving toward a comprehensive geriatric assessment and away from chronological age: *JACC* family series. JACC Adv.

[7] Shimura T., Yamamoto M., Kano S. (2017). Impact of the clinical frailty Scale on outcomes after transcatheter aortic valve replacement. Circulation.

[8] Goldfarb M., Lauck S., Webb J.G. (2018). Malnutrition and mortality in frail and non-frail older adults undergoing aortic valve replacement. Circulation.

[9] Généreux P., Piazza N., Alu M.C. (2021). Valve academic research consortium 3: updated endpoint definitions for aortic valve clinical research. Eur Heart J.

[10] Rockwood K., Song X., MacKnight C. (2005). A global clinical measure of fitness and frailty in elderly people. CMAJ.

[11] Dalhousie University, Faculty of Medicine, Division of Geriatric Medicine Clinical frailty scale. Halifax, Canada: Dalhousie University. http://geriatricresearch.medicine.dal.ca/clinical_frailty_scale.htm.

[12] Bouillanne O., Morineau G., Dupont C. (2005). Geriatric nutritional risk index: a new index for evaluating at-risk elderly medical patients. Am J Clin Nutr.

[13] Ministry of Health, Labour and Welfare The 22nd life tables, Ministry of health, labour and welfare; 2017, 2015. Tokyo, Japan: Director-general for statistics and information policy. http://www.mhlw.go.jp/.

[14] Noguchi M., Tabata M., Obunai K. (2021). Clinical outcomes of Transcatheter Aortic Valve Implantation (TAVI) in nonagenarians from the optimized catheter valvular intervention-TAVI registry. Catheter Cardiovasc Interv.

[15] Massussi M., Adamo M., Rosato S. (2022). Functional and metabolic frailty predicts mortality in patients undergoing TAVI: insights from the OBSERVANT II study. Eur J Intern Med.

[16] Delijani D., Li L., Rutkin B. (2023). Impact of age on outcomes after transcatheter aortic valve implantation. Eur Heart J Qual Care Clin Outcomes.

[17] Liang H., Li X., Lin X., Ju Y., Leng J. (2021). The correlation between nutrition and frailty and the receiver operating characteristic curve of different nutritional indexes for frailty. BMC Geriatr.

[18] Lohman M.C., Sonnega A.J., Resciniti N.V., Leggett A.N. (2020). Frailty phenotype and cause-specific mortality in the United States. J Gerontol A Biol Sci Med Sci.

[19] Norman K., Haß U., Pirlich M. (2021). Malnutrition in older adults-recent advances and remaining challenges. Nutrients.

[20] Ishizu K., Shirai S., Tashiro H. (2022). Prevalence and prognostic significance of malnutrition in older Japanese adults at high surgical risk undergoing transcatheter aortic valve implantation. J Am Heart Assoc.

[21] Riihiniemi M., Piuhola J., Niemelä M. (2024). Transcatheter aortic valve replacement in nonagenarians: a Finnish multicenter study. Am J Cardiol.

[22] Chopard R., Teiger E., Meneveau N. (2015). Baseline characteristics and prognostic implications of pre-existing and new-onset atrial fibrillation after transcatheter aortic valve implantation: results from the FRANCE-2 registry. JACC Cardiovasc Interv.

[23] James K., Jamil Y., Kumar M. (2024). Frailty and cardiovascular health. J Am Heart Assoc.

