# Supplementary material for: Long-Term Outcomes After Transcatheter Aortic Valve Replacement in Nonagenarians: Impact of Frailty and Malnutrition
Source: JACC Adv. 2026 Mar 25;5(3):102614. doi: 10.1016/j.jacadv.2026.102614 (PMC13352011; doi:10.1016/j.jacadv.2026.102614)
Supplement: Supplemental Material [file mmc1.docx]

**Supplementary Materials**

Supplementary Table 1. Sensitivity analysis: post-TAVR outcomes in patients ≥90 vs 80–89 years

Page 1

Supplementary Figure 1. Study flow and patient stratification Page 2

Supplementary Figure 2. Age-spline hazard ratios for all-cause mortality Page 3

Supplementary Figure 3. Causes of death by age group Page 4

Supplementary Figure 4. Relative survival in nonagenarians after TAVR Page 5

**Supplementary Table 1. Sensitivity analysis: post-TAVR outcomes in patients ≥90 vs 80–89 years**

| Endpoint | Unadjusted HR | P value | Adjusted HR | P value |
| --- | --- | --- | --- | --- |
| All-cause death | 1.54 (1.38–1.73) | <0.001 | 0.97 (0.82–1.15) | 0.720 |
| Cardiovascular death | 1.11 (0.93–1.32) | 0.245 | 0.84 (0.65–1.09) | 0.184 |
| Non-cardiovascular death | 1.66 (1.44–1.92) | <0.001 | 1.06 (0.84–1.34) | 0.605 |
| Hear failure readmission | 1.29 (1.05–1.59) | 0.016 | 1.41 (1.02–1.96) | 0.038 |
| Stroke | 0.81 (0.59–1.11) | 0.182 | 0.73 (0.47–1.15) | 0.177 |

HR = hazard ratio.

**Supplementary Figure 1**. **Study flow and patient stratification**

After exclusions, 4,623 patients were included. Patients were stratified by age: ≥90 years (n = 700) and <90 years (n = 3,923).

Abbreviation: TF = transfemoral.

**Supplementary Figure 2. Age-spline hazard ratios for all-cause mortality**

Restricted cubic spline Cox regression modeled age as a continuous variable, with 90 years as reference. Hazard ratios (solid line) with 95% confidence intervals (shaded area) remained stable between 70 and 85 years, increased after 87 years, and rose steeply beyond 90 years, indicating a continuous rather than stepwise risk increase.

**
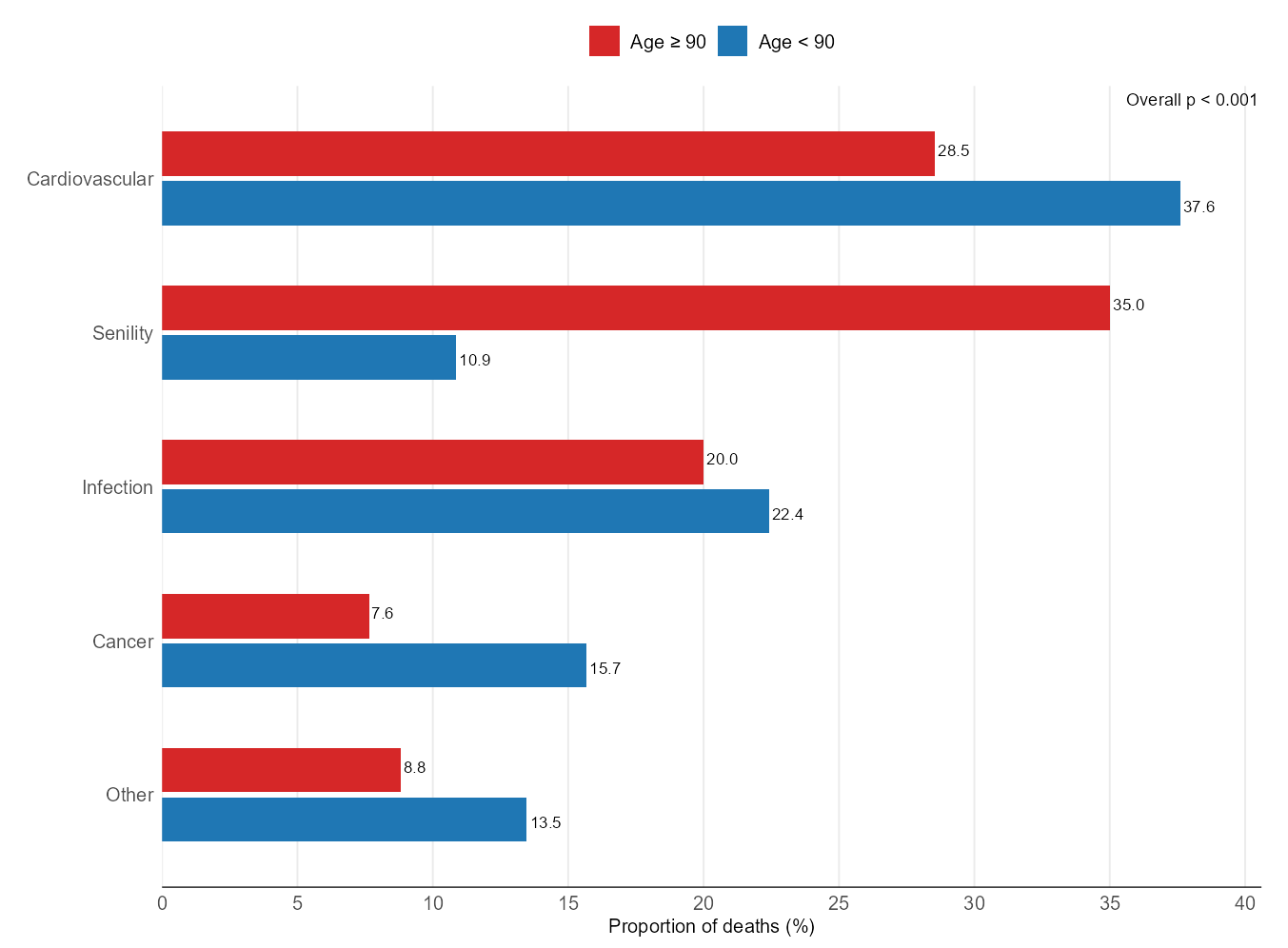
**

**Supplementary Figure 3**. **Causes of death by age group**

Horizontal bar chart comparing causes of death between patients aged ≥90 years (red, top) and <90 years (blue, bottom). The overall p value (upper right) is from a χ^²^ test comparing the 5-category distributions.

**Supplementary Figure 4. Relative survival in nonagenarians after TAVR**

Observed survival after transcatheter aortic valve replacement (TAVR) in patients aged ≥90 years (red line) is compared with age- and sex-matched expected survival from Japanese life tables (blue line). The green dashed line represents relative survival (observed/expected). Relative survival crossed 1.0 at approximately 2 years and remained ≥1.0 thereafter, indicating comparable or superior survival to the general population.

Abbreviation: TAVR = transcatheter aortic valve replacement
